# Outcome of a dedicated complex aortic surgery fellowship program

**DOI:** 10.3389/fsurg.2024.1404641

**Published:** 2024-07-31

**Authors:** Luis H. Arzola, Giuseppe Asciutto, Maysam Shehab, Anders Wanhainen, Kevin Mani

**Affiliations:** ^1^ Department of Surgical Sciences, Section of Vascular Surgery, Uppsala University, Uppsala, Sweden; ^2^ Department of Surgical and Perioperative Sciences, Surgery, Umeå University, Umeå, Sweden

**Keywords:** training, aortic surgery, fellowship, endovascular, research

## Abstract

**Introduction:**

Advancements in endovascular therapy, especially in complex aortic procedures, underscore the need for additional post-certification training. Dedicated post-residency vascular fellowship programs offer exposure to diverse cases, research opportunities, and professional networking. The study aims to describe and present outcomes from the complex aortic fellowship program at the Vascular Surgery Department of the Uppsala University Hospital.

**Methods:**

Nine former fellows who completed the aortic fellowship program at the Uppsala University Hospital from 2018 to 2023 were invited to take part in an anonymous 29-question survey through email. Demographic data, information about the existence of local aortic programs as well as on the types of aortic procedures performed were gained. The overall experience and impact of the fellowship were assessed using multiple interval scale questions, with a rating scale (1 excellent to 5 very poor). Finally, we provided the option to the participants to share additional feedback.

**Results:**

Median age of participants was 34 years (IQR, 30.5–36), with 44.4% being women (4/9). There was a significant variation in the number of publications produced during the fellowship, with an overall mean of 4 papers (IQR, 2–10). Regarding the long-term impact of the fellowship, 5/9 (55.6%) of the fellows have contributed to the implementation of a complex aortic program after finishing the fellowship, providing a broad range of complex aortic procedures. All fellows (9/9, 100%) stated that the quality of the fellowship was excellent. The clinical experience 7/9 (77.8%), the academic environment 7/9 (77.8%) and the research opportunities 7/9 (77.8%) together with the mentorship 9/9 (100%) and the work environment 8/9 (88.9%), were considered of most value among the fellows. In general, the survey participants agreed that the fellowship atmosphere was suitable for learning, 9/9 (100%), and that it had a positive impact on their current practice, 7/9 (77.8%). Currently, 5/9 (55.5%) of the fellows hold a position including academic involvement.

**Conclusions:**

There is a universal need for additional post-certification training. The current study showed that a balanced clinical and scientific exposure to complex aortic diseases is broadly welcomed among young vascular surgeons. The extension of the fellowship to cover other disciplines dealing with complex aortic procedures can be of value.

## Introduction

The 1970s marked a significant turning point in the effort to establish vascular surgery as a distinct subspecialty, gaining traction within the medical community. Subsequently, in 2005, vascular surgery was recognized as a separate and independent section within the Union of European Medical Specialists UEMS (1), and in 2006 the American Board of Surgery transitioned its vascular surgery certificate from a subspecialty to a specialty (primary) certification (2).

The following decade was spent implementing this new standard all over the world. At the moment, there are large differences in training in vascular surgery in various European countries. In Sweden, vascular surgery has become a primary specialty in 2015. Twenty-eight Swedish training centers are active in treating vascular diseases, with a different extent of complexity. The length of training years is at least five, while the number of trainees and of operations per center varies considerably.

With the rapid advancement of endovascular therapy, particularly in the complex aortic field, questions arise regarding the necessity of additional vascular surgery training post-primary certification. This aspect has been underlined by the results of an anonymous survey among 62 vascular surgeons who completed vascular surgery residency. In this cohort, 59% of the respondents reported a desire for additional complex aortic endovascular surgery training. Among them, 35% would have desired additional training in custom branched/fenestrated endografting (3).

The impact of a dedicated post-residency vascular fellowship program in a high-volume center of excellence extends to various aspects of a professional vascular surgeon's life. This encompasses exposure to a diverse range of surgical cases in terms of volume and complexity, engagement in academic and translational research, as well as opportunities for networking and lifelong mentorship and collaboration.

The aim of the current study is to describe and present the outcomes of a dedicated vascular fellowship program at the Vascular Surgery Department of the Uppsala University Hospital.

### The Uppsala aortic fellowship program

The Uppsala Aortic Fellowship (4) is an international, integrated clinical and research fellowship program committed to advancing expertise in the management of aortic pathologies among senior vascular trainees (Supplementary Material). Its primary objective is to provide a comprehensive and dedicated experience in the current management of complex aortic pathology. The fellowship program was first established in 2018 with the aim of recruiting international vascular fellows with an interest in the management of complex aortic pathology. The program runs over a period of 6–12 months.

The Department of Vascular Surgery at Uppsala University Hospital is a tertiary referral center for complex vascular pathologies and a leading research unit in vascular diseases. A dedicated staff of 5 senior consultants, two fellows, and three residents deal with all vascular pathologies with a special focus on aortic diseases. The Uppsala Aortic Center performs between 100 and 150 complex aortic reconstructions yearly. The unit is the primary initiator of several multicenter studies covering various aspects of aortic pathology.

### Program settings

The fellow actively participates in the comprehensive clinical management of patients with aortic disease, from planning to executing complex endovascular procedures. A crucial aspect of the fellowship program lies in the exposure to a range of standard and complex endovascular procedures designed for the treatment of aortic disease.

In conjunction with the fellowship curriculum, the fellow is mandated to become involved in a minimum of one aortic research project during the program. Furthermore, the presentation of research findings at an international scientific forum and subsequent publication in a peer-reviewed journal are essential components of this research commitment.

## Methods

We conducted a single-center study to assess the overall experience of the nine former fellows who completed the Aortic fellowship program at the Uppsala University Hospital from 2018 to 2023. The fellows were invited to take part in an anonymous survey through email, with information about the questionnaire and consent form included in the email.

### Questionnaire details

An anonymous 29-question survey was distributed to all vascular fellows (Supplementary Material). The first part of the questionnaire focused on demographic data, including age at the time of the fellowship, current practice, and academic background. Additionally, information about the existence of local aortic programs, as well as on the types of aortic procedures performed, was gained. The overall experience and impact of the fellowship were assessed using multiple interval scale questions, with a rating scale ranging from 1 to 5, where 1 indicated excellent and 5 indicated very poor. Finally, we provided the participants with the option to share additional feedback, suggestions, or insights aiming to possibly improve the settings of the fellowship program.

### Statistical analysis

Descriptive statistics were used, including frequency, mean, median, standard deviation, and interquartile (IQR) range measures. All statistical analyses were performed using SPSS Statistics for Windows version 28 (IBM Corp., Armonk, NY, USA).

## Results

**Figure 1:**
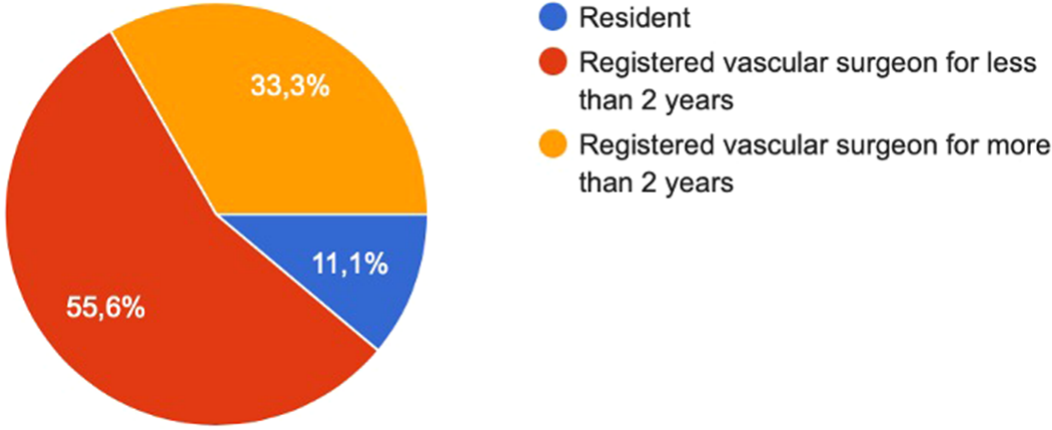
Academic position by the time of the fellowship.

A total of 9/9 (100%) aortic fellows completed the questionnaire. During their training, the median age of participants was 34 years [IQR, 30.5–36]. Gender distribution was balanced, with 44.4% of participants being women (4/9). Additionally, 5/9 (55.6%) were specialists within the first 2 years of practice, 3/9 (33.3%) were specialists with more than 2 years of practice, and 1/9 (11.1%) was still a resident (Figure 1).

### Surgical proficiency during the fellowship

**Figure 2:**
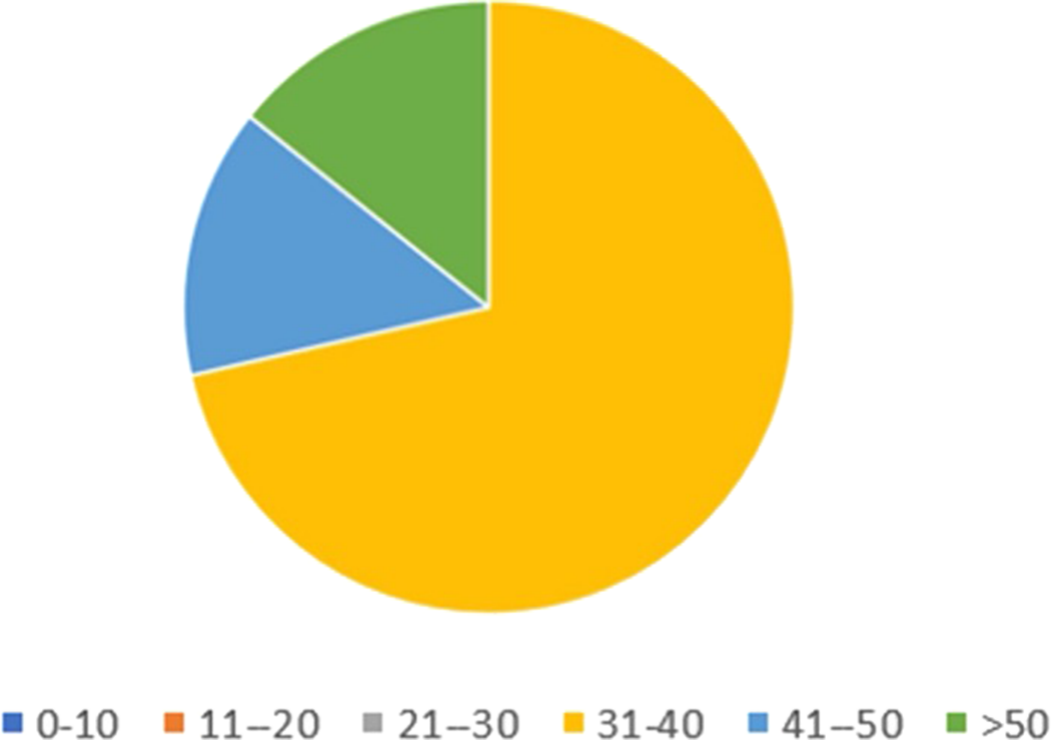
Total number of complex aortic cases planned by the fellow (see text for explanation).

**Figure 3:**
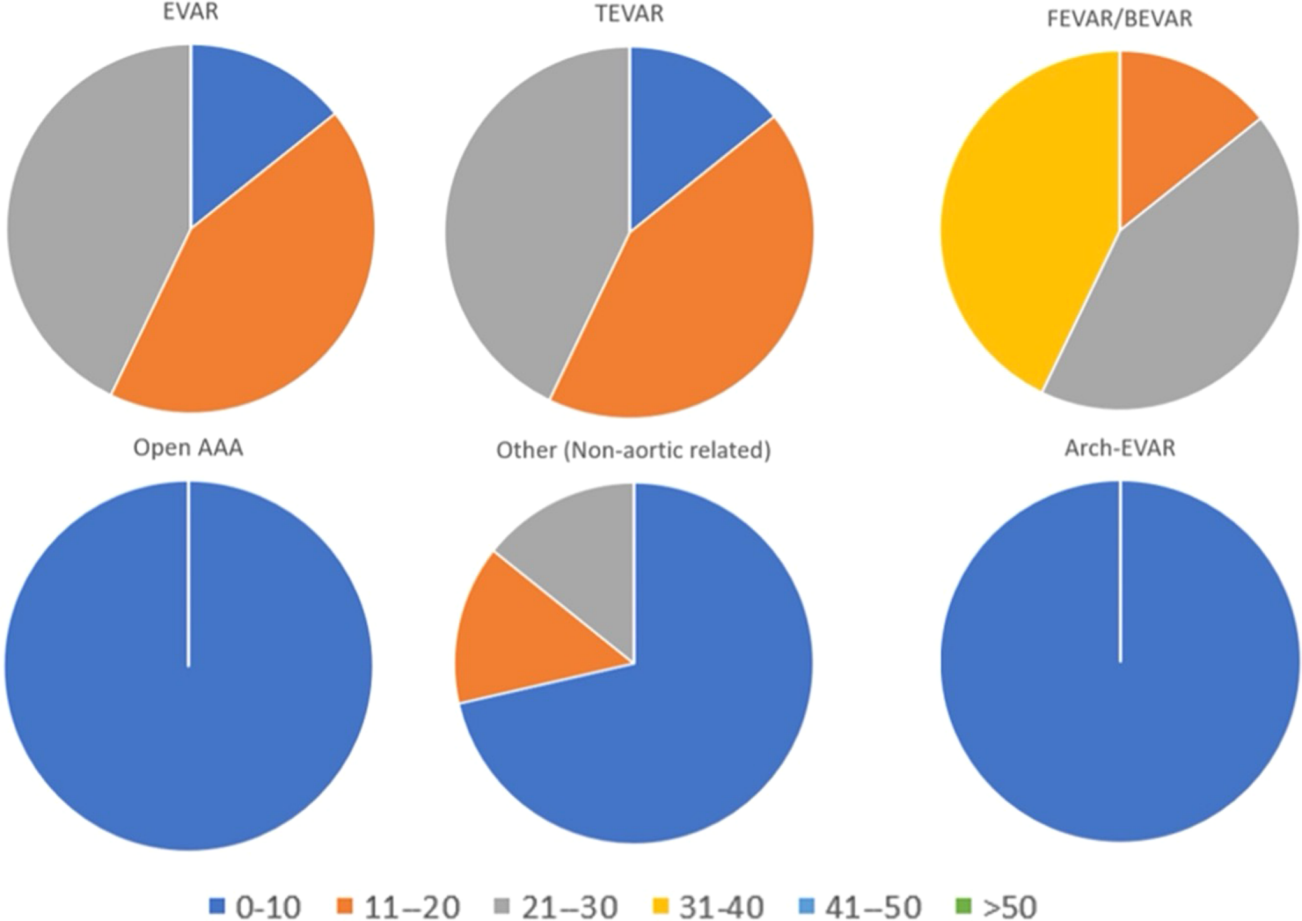
Total number of procedures assisted or performed as first operator, categorized by procedure type (see text for explanation).

Only seven out of nine fellows answered the questionnaire with regards to their surgical proficiency during the fellowship. During the fellowship 5/7 fellows (71.4%) planned between 31 and 40 (Figure 2) complex endovascular aortic repairs (EVARs), The type of procedures performed by all fellows was mostly endovascular, with 6/7 fellows (85.7%) who performed between 11 and 30 infrarenal EVARs and thoracic EVARs, while 6/7 of fellows (85.7%) performed between 21 and 40 fenestrated or branched EVARs (Figure 3).

### Current (post-fellow) practice

**Figure 4:**
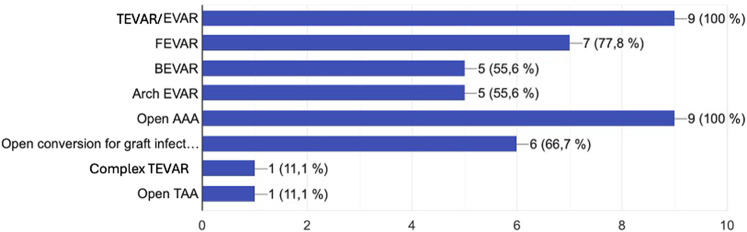
Type of aortic procedures currently performed by Uppsala aortic fellowship participants as independent consultants post-fellowship.

Practice patterns of former fellows were classified based on academic position, practice setting, number of publications, level of academic appointment, and type of aortic procedures performed at their institutions. Overall, 4/9 (44.4%) hold a hospital-based position, 3/9 (33.3%) hold an academic-based position, and 2/9 (22.2%) hold both. Regarding the level of academic appointment, 3/9 (33.3%) had been registered as a PhD student at Uppsala University during or after the fellowship. There was a significant variation in the number of publications produced during the fellowship, with an overall mean of 4 papers [IQR, 2–10]. Regarding the long-term impact of the fellowship, 5/9 (55.6%) of the fellows have contributed to the implementation of a complex aortic program after finishing the fellowship, providing a broad range of complex aortic procedures (Figure 4).

### Fellowship assessment

**Figure 5:**
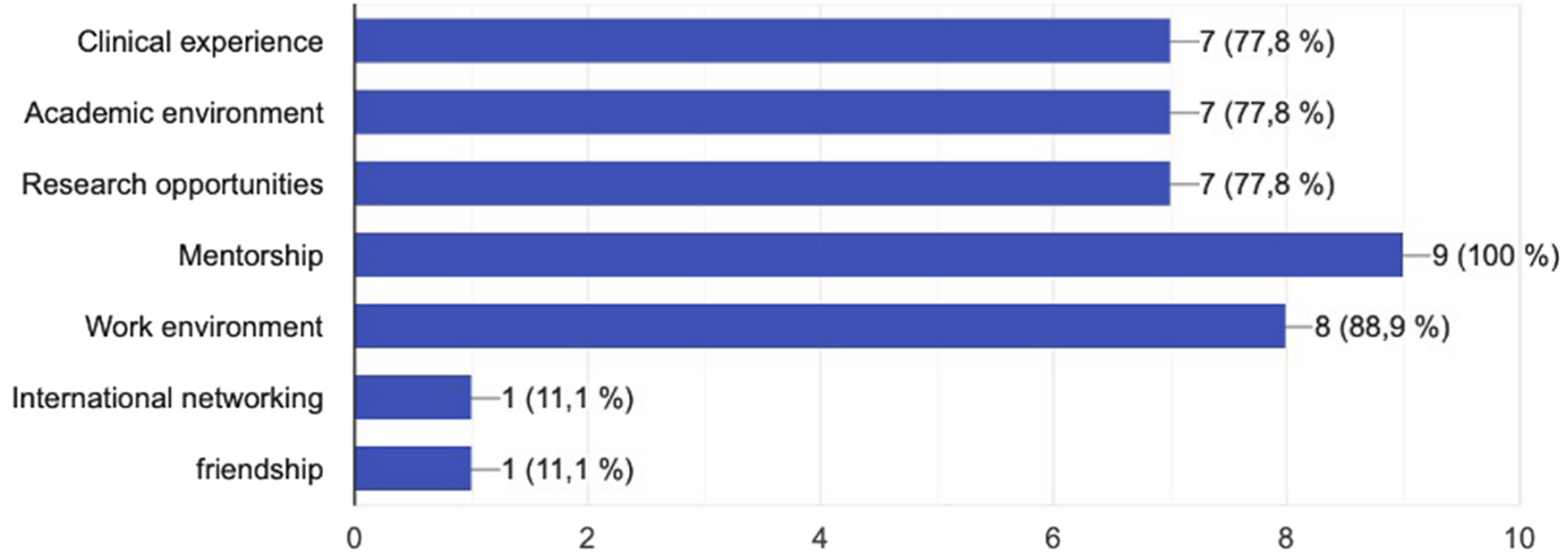
Most valuable aspects of the fellowship program.

All, i.e., 9/9 (100%) former fellows stated that the quality of the fellowship was excellent, and 8/9 (88.9%) thought that it exceeded their expectations. Several features were evaluated to establish the most valuable aspect of the fellowship (Figure 5). The clinical experience 7/9 (77.8%), the academic environment 7/9 (77.8%) and the research opportunities 7/9 (77.8%) together with the mentorship 9/9 (100%) and the work environment 8/9 (88.9%), were considered of most value among the fellows.

### Career impact

**Figure 6:**
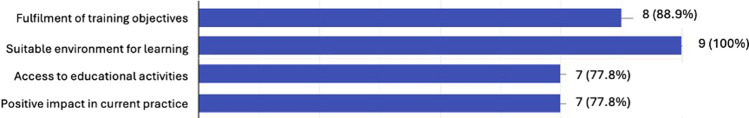
Aortic fellowship program characteristics.

The majority of the fellows considered they met the stated training objectives from the aortic fellowship program, 8/9 (88.9%), with significantly fair access to educational opportunities as well as notable networking and professional development opportunities in 7/9 (77.8%) cases. In general, the survey participants agreed that the fellowship atmosphere was suitable for learning, 9/9 (100%), and that it had a positive impact on their current practice, 7/9 (77.8%) (Figure 6).

### Feedback

**Figure 7:**
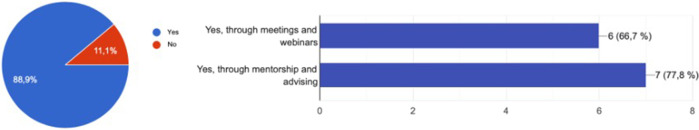
Continuous communication between previous fellows and the program staff.

The fellows kept contact with the program staff, with 8/9 (88.9%) citing academic purposes such as mentoring and advising, as well as engaging in social activities, even several years after completing the fellowship (Figure 7). Moreover, all participants 9/9 (100%) expressed their willingness to recommend or have already recommended the fellowship program to other colleagues. Finally, the suggestions included organizing an annual alumni meeting, increasing collaboration with the cardiac surgery team for exposure to open thoracic aortic cases, and updating the webpage.

## Discussion

The importance of continuing medical education (CME) is universally accepted. Concerns about the content, funding, and quality of *ad hoc* compulsory institutionalized programs have been raised. Besides local grand rounds and case discussions, national and international meetings can help health practitioners to maintain, develop, or increase their expertise and skills. However, the medical community agrees on the fact that there is no better way than hands-on training in order to update and extend overall theoretical and practical competence. Therefore, questions arise regarding the necessity of additional training post-primary certification.

The most recent guidelines of the European Society for Vascular Surgery on the management of abdominal aorto-iliac artery aneurysms (5) recommend a set minimum surgical volume for aortic centers based on the current evidence of a volume-outcome relationship in complex aortic repair. However, it is also suggested to adjust for local circumstances, political implications, geographic, and epidemiological factors when arguing for the centralization of complex aortic procedures. In this sense, additional training post-primary certification can help in updating and extending overall theoretical and practical skills in regions where complex aortic repair is not practiced. With this aim, post-graduate programs have been developed during the last decades.

Surgical fellowships in cardiovascular diseases are currently running (6, 7). Only a few of them are focused on the endovascular treatment of complex aortic diseases.

The Uppsala Aortic Fellowship is an international, integrated clinical and research fellowship program aiming to improve knowledge and expertise in the management of complex aortic pathologies.

An active participation in the comprehensive clinical management of patients with aortic disease is guaranteed through continuous exposure to a range of standard and complex endovascular procedures. Performing research and presenting research findings nationally and internationally, as well as publication in peer-reviewed journals, are essential components of the fellowship program.

The results of the current survey show a generally positive acceptance of the hybrid concept of the Uppsala Aortic Fellowship, covering both clinical and scientific aspects regarding complex aortic diseases. In particular, the clinical experience, the academic environment, and the research opportunities together with the mentorship, were considered of most value among the fellows.

**Figure 8:**
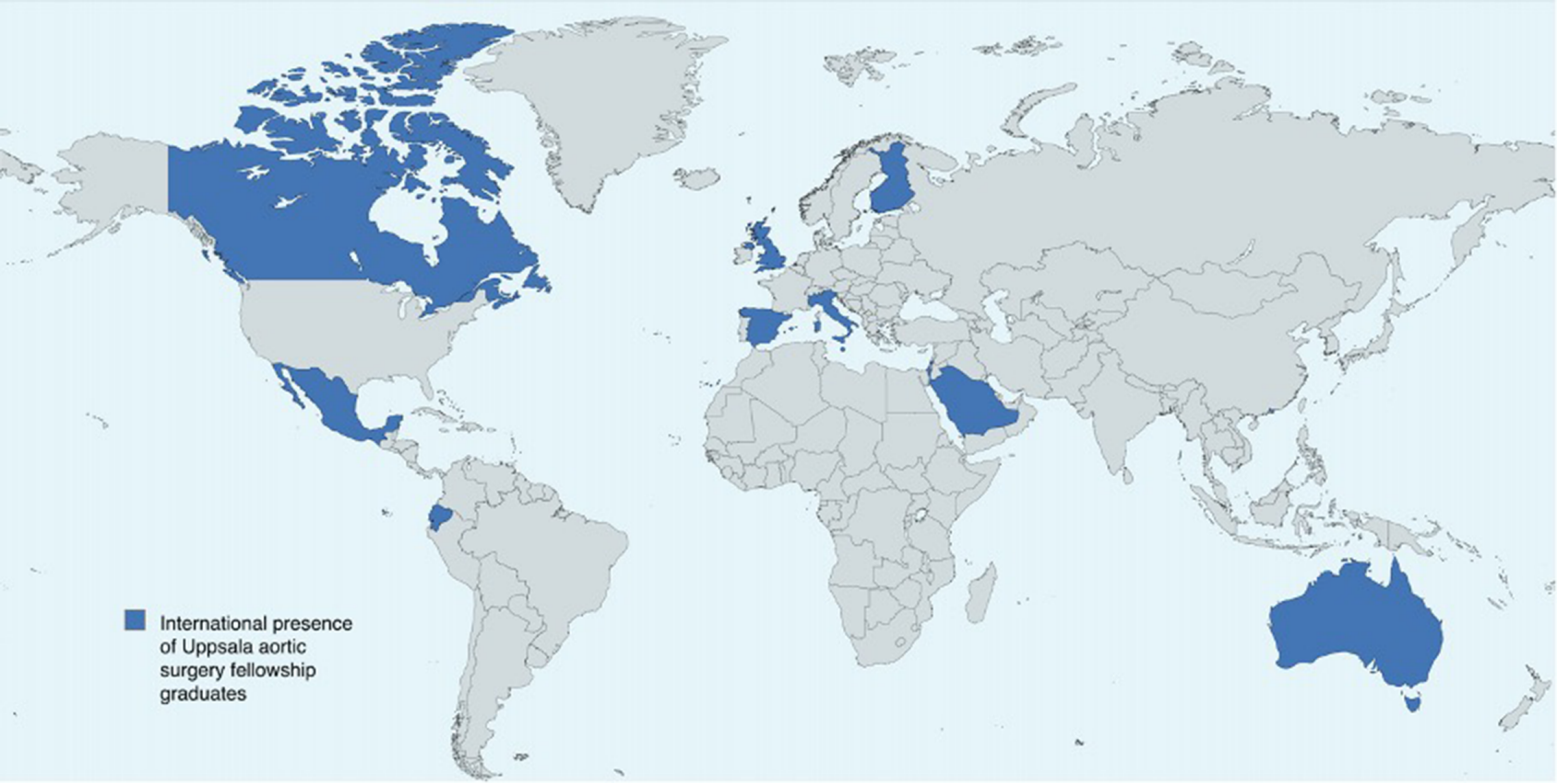
International presence of Uppsala aortic fellowship graduates.

The majority of the respondents agreed that the fellowship atmosphere was suitable for learning and that it had a positive impact on their current practice. In particular, the fellowship has been recognized for its contribution to the implementation of complex aortic programs around the world (Figure 8).

Aware of the fact that the Uppsala Aortic Fellowship has potential for improvement, we asked for suggestions on how to further increase the quality of the program. In particular, increasing collaboration with the cardiac surgery team in order to be exposed to open thoracic aortic cases is one of the suggestions that will be implemented in the future.

## Conclusions

Maintaining and developing knowledge and practical skills is paramount in modern health care. Postgraduate fellowships may help in training future generations of healthcare providers.

For the case of vascular surgery, the current study showed that a balanced clinical and scientific exposure to complex aortic diseases is considered of value among young vascular surgeons. The extension of the fellowship to cover other disciplines dealing with complex aortic procedures can be of value.

## Data Availability

The data analyzed in this study is subject to the following licenses/restrictions: the dataset is an anonymous survey. Requests to access these datasets should be directed to Luis H Flores, lhaflores@hotmail.com.
