# Supplementary material for: Outcome of a dedicated complex aortic surgery fellowship program
Source: Front Surg. 2024 Jul 31;11:1404641. doi: 10.3389/fsurg.2024.1404641 (PMC11322339; doi:10.3389/fsurg.2024.1404641)
Supplement: Supplementary file 1 [file Datasheet1.pdf]

	Fellows								
**Questions**	**1**	**2**	**3**	**4**	**5**	**6**	**7**	**8**	**9**
Overall, how would you rate the quality of the fellowship training? 1 very poor, 2 poor, 3 acceptable, 4 good, 5 very good	5	5	5	5	5	5	5	5	5
Have you been able to apply the skills and knowledge gained during the fellowship to your current role? 1 often, 2 sometimes, 3 occasionally, 4 rarely, 5 never	1	1	2	1	1	1	1	1	2
The Aortic Fellowship program met the stated training objectives. 1 strongly agree, 2 agree, 3 neutral, 4 disagree, 5 strongly disagree	1	1	1	1	2	1	1	1	1
I had a mentor(s) who supported and encouraged my professional development during my fellowship. 1 strongly agree, 2 agree, 3 neutral, 4 disagree, 5 strongly disagree	1	1	1	1	1	1	1	1	1
My access to opportunities was fair and equitable compared to my peers. 1 strongly agree, 2 agree, 3 neutral, 4 disagree, 5 strongly disagree	1	1	2	1	3	1	1	1	1

The fellowship atmosphere was suitable for learning. 1 strongly agree, 2 agree, 3 neutral, 4 disagree, 5 strongly disagree	1	1	1	1	1	1	1	1	1
The Aortic Fellowship has positively impacted my career development. 1 strongly agree, 2 agree, 3 neutral, 4 disagree, 5 strongly disagree	1	2	2	1	1	1	1	1	1
The Aortic Fellowship provided me with opportunities for networking and professional development. 1 strongly agree, 2 agree, 3 neutral, 4 disagree, 5 strongly disagree	1	2	2	1	1	1	1	1	1
