# Supplementary material for: Outcome of a dedicated complex aortic surgery fellowship program
Source: Front Surg. 2024 Jul 31;11:1404641. doi: 10.3389/fsurg.2024.1404641 (PMC11322339; doi:10.3389/fsurg.2024.1404641)
Supplement: Supplementary file 2 [file Datasheet2.pdf]

Uppsala Aortic Fellowship Scientific programme

The Uppsala Aortic Fellowship (UAF) is a combined clinical and research fellowship program dedicated to promoting knowledge in management of aortic pathologies among senior vascular trainees.

The fellowship was initiated based on a grant from Cook. The UAF program has been an extremely popular fellowship program, which has attracted significant local, national and international interest. Applications for the past positions have been received from vascular surgeons, interventional radiologists and cardiothoracic surgeons from Europe, USA, South America, Australia, Asia and Africa. The programme has enabled several aortic surgeons and interventionalists to participate in the Uppsala Aortic Fellowship, all of whom have gone on to consultant vascular surgical positions in prestigious academic vascular institutions around the world, with significant aortic surgical activity. Several international collaborative research and development projects in the field of aortic surgery have been initiated thanks to this fellowship program. All fellows have reached significant experience in planning and performing standard and complex endovascular aortic procedures, including fenestrated and branched endovascular aortic repair.

Based on this success, the current application refers to a continuation of the fellowship. We previously applied for the 2023 funding, which was approved for one semester, and herein would like to extend the application for autumn 2023 and spring 2024.

Fellowship program design

The vascular surgical department at Uppsala University Hospital is a tertiary referral centre for complex aortic pathology, and a leading research unit in studies of aortic disease.

The UAF program is aimed for senior vascular trainees or early post-graduate specialists in vascular surgery with a specific interest to gain in depth experience in modern management of aortic pathology. Two 6-months fellowship positions are announced, with a combined clinical and research profile. The successful applicant is expected to spend 75% of the fellowship period in clinical service, and 25% in pursuing a specific research project on treatment of aortic disease with endovascular techniques (project directly related to the fellowship program goal).

In addition to the full fellowship program, the UAF offers the possibility for two short term fellowship periods of 4 weeks.

The fellowship trainee is involved in the clinical management of patients with aortic disease, including patient assessment, operative planning, endovascular and open surgical procedures, and follow-up management. Exposure to standard and complex endovascular procedures for aortic disease is a key component of the fellowship program.

As part of the fellowship program, the trainee is involved in a dedicated research project in aortic disease. The academic unit pursuits several aortic projects including studies of aortic disease pathophysiology, prevention and surgical management, as well as studies of novel surgical and endovascular techniques. Opportunity to present research projects in national and international scientific forum is offered.

Objectives

To promote in depth understanding of modern management of aortic pathology, including aspects on screening for aortic disease, medical management, imaging, open and endovascular surgical treatment, and follow-up

To introduce the fellow to current challenges in further improving the care of patients with aortic disease, and to introduce the fellow to research in aortic disease

Outcome

After completion of the fellowship program, the successful participant is equipped to continue a career path in aortic surgery, with a combined academic and clinical profile.

Fellowship period

Two six-months fellowships, autumn 2023 and spring 2024. Additionally, two 4-weeks short-term fellows are accepted throughout the year.

Formal requirements

The successful applicant should be a senior vascular trainee or in early post-graduate period after completion of vascular surgical training. Basic endovascular experience is required. Academic experience is not mandatory, but academic interest is expected.

The program is primarily aimed at international applicants. Fluency in English is preferred, but knowledge in Swedish is not required. Registration with the Department of Health in order to practice as physician in Sweden is beneficial.
