# Supplementary material for: Outcome of a dedicated complex aortic surgery fellowship program
Source: Front Surg. 2024 Jul 31;11:1404641. doi: 10.3389/fsurg.2024.1404641 (PMC11322339; doi:10.3389/fsurg.2024.1404641)
Supplement: Supplementary file 3 [file Datasheet3.pdf]

Uppsala Aortic Fellowship Questionnaire

* Indicates required question

1. **How old were you when you started the fellowship?** *

   ____

2. **Which position did you have when you started the fellowship?**

   Mark only one oval.

   - ◯ Resident
   - ◯ Registered vascular surgeon for less than 2 years
   - ◯ Registered vascular surgeon for more than 2 years

3. **How would you describe your current workplace? You can select more than one option.** *

   Check all that apply.

   - ☐ Hospital (clinical practice)
   - ☐ Academic institution
   - ☐ Other: ________________________

4. **Have you published in peer-reviewed journals during or after finishing your fellowship?** *

   Mark only one oval.

   - ◯ Yes
   - ◯ No

5. **If yes, please specify how many papers total and how many with the Uppsala research group**

6. **Have you been registered as PhD student during/after the fellowship?** *

Mark only one oval.

- Yes, at Uppsala University
- Yes, at home
- No, I have not

7. **Have you contributed or implemented any new programs at your hospital since you finished your fellowship?**

Mark only one oval.

- Yes
- No

8. **If yes, please provide examples**

9. **Please indicate the total number of cases you participated in as the main or assisting operator during your fellowship.**

Check all that apply.

EVAR	0-10 ☐	11-20 ☐	21-30 ☐	31-40 ☐	41-50 ☐	>50 ☐
TEVAR	0-10 ☐	11-20 ☐	21-30 ☐	31-40 ☐	41-50 ☐	>50 ☐
FEVAR/BEVAR	0-10 ☐	11-20 ☐	21-30 ☐	31-40 ☐	41-50 ☐	>50 ☐
Arch EVAR	0-10 ☐	11-20 ☐	21-30 ☐	31-40 ☐	41-50 ☐	>50 ☐
Open AAA	0-10 ☐	11-20 ☐	21-30 ☐	31-40 ☐	41-50 ☐	>50 ☐
Other	0-10 ☐	11-20 ☐	21-30 ☐	31-40 ☐	41-50 ☐	>50 ☐

10. **Please indicate the total number of complex aortic cases you planned during your fellowship.**

0-10 ☐ 11-20 ☐ 21-30 ☐ 31-40 ☐ 41-50 ☐ >50 ☐

11. **Please select from the list below which procedures are currently performed at your institution?**

Check all that apply.

☐ EVAR
☐ FEVAR
☐ BEVAR
☐ Arch EVAR
☐ Open AAA
☐ Open conversion for graft infection
☐ Other: ______________________

12. **Overall, how would you rate the quality of the fellowship training?** *

1 very poor, 2 poor, 3 acceptable, 4 good, 5 very good

Mark only one oval.

1	2	3	4	5
◯	◯	◯	◯	◯

13. **To what extent did the clinical fellowship training meet your expectations based on the goal and objectives identified at the start of your fellowship?**

Mark only one oval.

- ◯ Exceeded my expectations
- ◯ Met my expectations
- ◯ Did not meet my expectations
- ◯ I did not complete my clinical fellowship

14. **How did the fellowship prepare you for the challenges of practicing specialist aortic surgery care?**

Mark only one oval.

- Very well-prepared
- Somewhat prepared
- Not well-prepared
- Not applicable

15. **Which of the following did you provide as part of your fellowship? Check all that apply.** *

Check all that apply.

- [ ] Direct patient care
- [ ] Academic lectures
- [ ] Complex aortic case planning
- [ ] Research
- [ ] Other: ______________________________

16. **Did you present any of your research projects started in Uppsala during the fellowship?** *

Check all that apply.

- [ ] Yes, at an international meeting
- [ ] Yes, at the Uppsala Vascular Research Network
- [ ] No, but I will present something in the near future
- [ ] No

17. **What was the most valuable aspect of the fellowship training? You can select more than on option.**

Check all that apply.

- [ ] Clinical experience
- [ ] Academic environment
- [ ] Research opportunities
- [ ] Mentorship
- [ ] Work environment
- [ ] Other: ______________________

Carrer impact

To what extent do you agree or disagree with each of the following statements?

18. **Have you been able to apply the skills and knowledge gained during the fellowship to your current role?**

1 often, 2 sometimes, 3 occasionally, 4 rarely, 5 never

Mark only one oval.

1	2	3	4	5
◯	◯	◯	◯	◯

19. **The Aortic Fellowship program met the stated training objectives.** *

1 strongly agree, 2 agree, 3 neutral, 4 disagree, 5 strongly disagree

Mark only one oval.

1	2	3	4	5
◯	◯	◯	◯	◯

20. **I had a mentor(s) who supported and encouraged my professional development during my fellowship.**

1 strongly agree, 2 agree, 3 neutral, 4 disagree, 5 strongly disagree

Mark only one oval.

1	2	3	4	5
◯	◯	◯	◯	◯

21. **My access to opportunities was fair and equitable compared to my peers.** *

1 strongly agree, 2 agree, 3 neutral, 4 disagree, 5 strongly disagree

Mark only one oval.

1	2	3	4	5
◯	◯	◯	◯	◯

22. **The fellowship atmosphere was suitable for learning.** *

1 strongly agree, 2 agree, 3 neutral, 4 disagree, 5 strongly disagree

Mark only one oval.

1	2	3	4	5
◯	◯	◯	◯	◯

23. **The Aortic Fellowship has positively impacted my career development.** *

1 strongly agree, 2 agree, 3 neutral, 4 disagree, 5 strongly disagree

Mark only one oval.

1	2	3	4	5
◯	◯	◯	◯	◯

24. **The Aortic Fellowship provided me with opportunities for networking and professional development.**

1 strongly agree, 2 agree, 3 neutral, 4 disagree, 5 strongly disagree

Mark only one oval.

1	2	3	4	5
◯	◯	◯	◯	◯

25. What personal or professional achievement, if any, is facilitated or impacted by completing th fellowship program?

____

____

____

____

____

Feedback

26. **Have you remained connected with the fellowship program since completing it?** *

Check all that apply.

- [ ] Yes, through meetings and webinars
- [ ] Yes, through mentorship and advising
- [ ] No, but I would like to stay connected
- [ ] No, I am not interested

27. **Would you recommend the Aortic Fellowship training at the University of Uppsala to your colleagues?**

Mark only one oval.

- ( ) Definitely/ have done it already
- ( ) Very likely
- ( ) Somewhat likely
- ( ) Not likely
- ( ) Definitely not

28. **What suggestions do you have for improving the fellowship program? Are there any additional training or support areas that would benefit future fellows?**

29. **Is there anything else you want to share about your experience with the fellowship program Please feel free to share any additional feedback, suggestions, or insights.**
